# Memory guidance of value-based decision making at an abstract level of representation

**DOI:** 10.1038/s41598-020-78460-6

**Published:** 2020-12-09

**Authors:** Anna Liashenko, Aslan S. Dizaji, Lucia Melloni, Caspar M. Schwiedrzik

**Affiliations:** 1 Neural Circuits and Cognition Lab, European Neuroscience Institute Göttingen – A Joint Initiative of the University Medical Center Göttingen and the Max Planck Society, Grisebachstraße 5, 37077 Göttingen, Germany; 2 International Max Planck Research School Neurosciences at the Georg August University Göttingen, Grisebachstraße 5, 37077 Göttingen, Germany; 3 Department of Neuroscience, Max Planck Institute for Empirical Aesthetics, Grüneburgweg 14, 60322 Frankfurt am Main, Germany; 4 Department of Neurology, New York University School of Medicine, 223 East 34th Street, New York, NY 10016 USA; 5 Perception and Plasticity Group, German Primate Center – Leibniz Institute for Primate Research, Kellnerweg 4, 37077 Göttingen, Germany

**Keywords:** Human behaviour, Decision, Learning and memory, Object vision

## Abstract

Value-based decisions about alternatives we have never experienced can be guided by associations between current choice options and memories of prior reward. A critical question is how similar memories need to be to the current situation to effectively guide decisions. We address this question in the context of associative learning of faces using a sensory preconditioning paradigm. We find that memories of reward spread along established associations between faces to guide decision making. While memory guidance is specific for associated facial identities, it does not only occur for the specific images that were originally encountered. Instead, memory guidance generalizes across different images of the associated identities. This suggests that memory guidance does not rely on a pictorial format of representation but on a higher, view-invariant level of abstraction. Thus, memory guidance operates on a level of representation that neither over- nor underspecifies associative relationships in the context of obtaining reward.

## Introduction

Many value-based decisions need to be taken in situations where it is not immediately apparent from currently available evidence alone which choice is better. In such situations, querying memory for past experiences can inform the decision-making process^1^. Memory-guidance can occur on the basis of similarity, i.e., memories of stimuli that are physically similar to the currently encountered one guide choice. Or, memory guidance occurs by association, i.e., events previously linked to the currently available evidence through experience are leveraged for decision-making. This frees the decision maker from relying only on memories directly associated, e.g., with reward. A classic example for the latter is the phenomenon of “sensory preconditioning”^2^: here, one first learns a value-free association between two stimuli, e.g., that person A is related to person B. Subsequently, person B is associated with reward. Value then spontaneously spreads by association from person B to person A, leading a decision maker to choose person A, although A was never directly associated with reward. But how specific is this form of memory guidance? Does value spread also to another, perhaps similar person, or does associative memory-guidance fail if person A is encountered under different circumstances?

A critical determinant of the specificity or generality of memory guidance is the format of the involved memory representation. This format could be abstract or highly specific. For example, memory guidance could occur at the level of conceptual, high-level representations about individuals, which would entail information along multiple dimensions, such as the name, occupation, and appearance of person A. Pictorial fidelity, on the other hand, would likely be discarded. Such abstract representations generalize efficiently, prevent overload^3^, and may be the prevalent form of representation in long-term memory^4^. In fact, it has been suggested that associative memory guidance of decision-making occurs at the level of categories^5^, i.e., with representations that contain representative information about category members. On the other hand, abstract representations may be error-prone, leading to false memories, and could spread too far, leading to overgeneralization. This can be observed clinically in anxiety disorders^6^ or socially in the “spreading attitude effect”, where negative valuations of a person spread to its associates by mere association^7^.

Alternatively, guidance by memory could rely on highly specific representations. Many forms of memory show high specificity, often down to pictorial detail. Visual recognition memory for objects stores pictorial detail with very high capacity^8^. Visual episodic memories preserve the original perspective in which the experience was made^9,10^. Similarly, rodents in a water maze approach a hidden support from a remembered direction^11^, which suggests that they use view-specific representations available in spatial memory. Such specific memory representations are computationally flexible, can prevent overgeneralization but put high demands on storage, and may be too narrow to efficiently guide decision-making beyond the context in which they were originally acquired. Indeed, sensory preconditioning with aversive tastes suggests that no memory guidance takes place when choice occurs in a different context than where the associations were originally acquired^12^, pointing at high specificity of the underlying memory.

In many memory systems, both abstract, conceptual and specific, pictorial representations coexist. For example, in visual working memory, two separate forms of representation retain information in a view-specific and view-invariant format, respectively^13^. Similarly, in episodic memory, detailed and more abstract representations coexist with different temporal decay functions and availability for retrieval^4^. Here, we investigate what kind of memory representation, abstract or pictorial, guides decision making. To this end, we investigate transfer of value within a distinct category, faces, using a sensory preconditioning paradigm^14^. Faces are easy to remember^15^, high-dimensional yet well-defined visual stimuli. Faces are represented both in pictorial and in abstract form at different stages of processing: on the one hand, face recognition memory for unfamiliar persons is highly specific, showing a strong decline in recognition performance when basic visual features such as head orientation are changed between study and test phase, suggesting a pictorial format of representation^16^. On the other hand, view-invariant representations of faces exist for object recognition in the visual system^17^ and even more abstract, conceptual representations have been found in the medial temporal lobe system, including the hippocampus^18^. To dissociate which format of representation is used for memory-guided decision-making, we focus on two specific dimensions, head orientation, which is relevant for image-specific encoding of information, and facial identity, which entails abstraction beyond pictorial image features. Specifically, we address whether acquired value from a rewarded picture of person B spontaneously spreads not only to the previously associated picture of person A (as in classic sensory preconditioning), but also to new pictures (views) of person A that were never shown during learning. We address two main questions:

1. Is memory-guidance specific enough to guide decisions within a class of stimuli (i.e., about different identities) as suggested by^7,19^, or does it occur on the level of category, as discussed by^5^?
2. Is memory-guidance pictorial (i.e., view-specific) or abstract (i.e., view-invariant)?

In addition, we assess to what extent memory guidance is driven by factors intrinsic to the image (i.e., image-computable memorability) and/or factors intrinsic to the observer (i.e., face episodic memory capacity), as we all as the roles of exposure and awareness.

## Results

**Figure 1:**
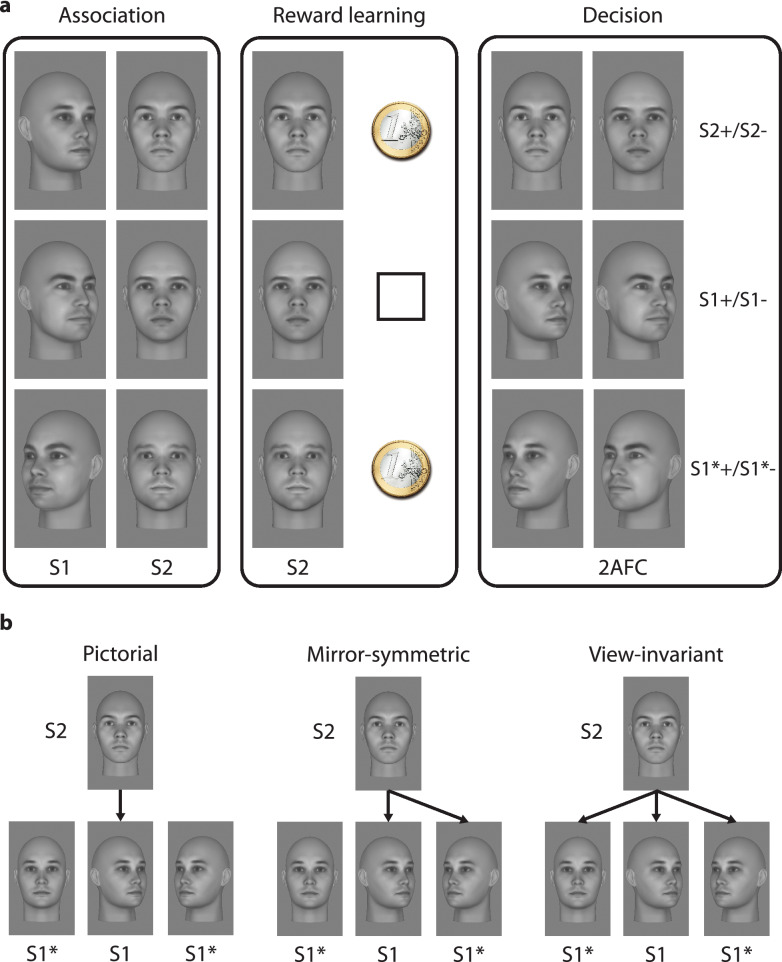
Paradigm and possible gradients of generalization. (**a)** The preconditioning paradigm has three phases: (1) association learning, (2) reward learning, and (3) decision-making. In the association phase, participants are exposed to six pairs of faces in a statistical learning paradigm. This establishes associations between S1 and S2. In the pursuant reward phase, participants learn through conditioning that half of the S2 are associated with monetary reward (S2+), whereas the other S2 stimuli predict a neutral outcome (S2−). No S1 are shown at this stage. In the final decision phase, participants perform a 2-alternative forced choice (2AFC) task to decide between two stimuli (S1+ vs. S1− or S2+ vs. S2−) for a possible monetary win. In addition, we present S1 stimuli with new head orientations (S1*+ vs. S1*−). The tendency to choose S1+ over S1− indicates transfer of reward from S2 to S1, and thus memory guidance of decision making. If subjects also chose S1*+ over S1*−, this indicates automatic generalization from S1 to S1*. (**b)** Memory guidance of decision making by association could be specific for the images shown during the association phase, i.e., memory guidance would occur on a basis of a pictorial format of representation. Alternatively, if there is generalization to new views, this could either follow a narrow gradient of generalization to mirror-symmetric versions of the same head orientation as in the original association; or, memory guidance could rely on view-invariant associations, generalizing to all head orientations. Faces in a and b are taken from “25 White Faces Manipulated on Trustworthiness (Steps of 1 SD)” data set from the Social Perception Lab (http://tlab.princeton.edu/databases/), and are reproduced with permission.

To address these questions, we conducted a sensory preconditioning experiment in 30 healthy volunteers (mean age 26.9 ± 5.7 years, 17 female, all right-handed) following procedures previously established by Wimmer and Shohamy^14^. This experiment consists of three phases: association learning, reward learning, and decision-making (Fig. 1a).

During the association phase, participants were exposed to 12 greyscale, computer-generated images depicting distinct male, Caucasian identities without hair and with neutral facial expression. Images were tightly controlled for low-level features (luminance), equated for trustworthiness, and titrated for likeability per subject (see Methods). The images were grouped into six pairs, presented in temporal succession (S1, S2) to establish associations between stimuli, while subjects did a cover task that drew attention to the stimuli but not to the associations themselves (see Methods)^20^. All S2 faces were frontal views, while S1 faces were either frontal (2), left profile (2), or right profile (2) views. Importantly, subjects were only exposed to one view per identity. To assess whether the amount of exposure during the association phase had an effect on memory guidance, one group of subjects (n = 15, mean age 26.6 ± 7.15 years, 8 female) was trained for one block (12 trials per pair) and one group (n = 15, mean age 25.1 ± 3.85 years, 9 female) for four blocks (48 trials per pair).

During reward learning, participants underwent conditioning, whereby half of the S2 stimuli were paired with monetary reward (S2+), while the remaining S2 were followed by a neutral outcome (S2−) (see Methods). No S1 stimuli were shown in this phase.

In the decision-making phase, we used a 2-alternative forced choice (2AFC) task to assess transfer of reward from S2+ to S1 (see Methods). Participants were asked to select which of two simultaneously presented stimuli, either S2+ and S2− or S1 previously paired with S2+ (S1+) and S1 previously paired with S2− (S1−), was more likely to lead to monetary reward. To determine whether transfer of value from S2+ was bound to the specific S1 images to which they were previously associated, or generalized, we also presented new S1 stimuli depicting the same identities but different head orientations during the decision-making phase (S1*). On these trials, subjects had to choose between new views of S1+ and S1−.

Finally, we performed a test for explicit memory of associations and conducted the Cambridge Face Memory Test (CFMT)^21^ with every participant to independently assess episodic memory capacity for faces (see Methods).

### Transfer of reward from S2 to S1

**Figure 2:**
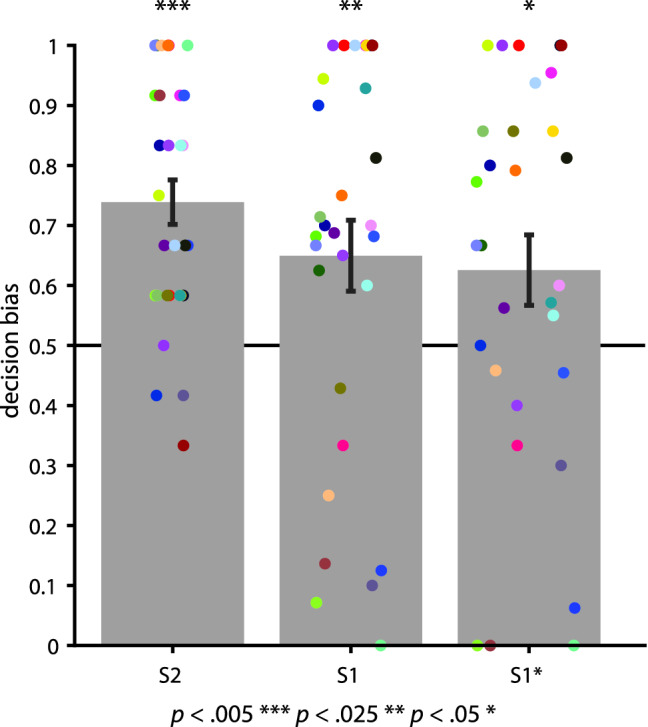
Average decision bias. During the decision-making phase, subjects chose S2+ over S2− (mean S2+ bias 73.89% ± 3.7 SEM, t_(29)_ = 6.4572, *p* < 0.001, g = 1.1482), which confirmed that conditioning during the preceding reward learning phase was successful. Subjects also showed memory guidance of decision making by choosing S1+ over S1− significantly more often than expected by chance (mean S1+ bias 64.96% ± 5.92 SEM, t_(29)_ = 2.5282, *p* = 0.0172, g = 0.4616). Memory guidance generalized to new views of the same facial identities, as evidenced by subjects choosing S1*+ over S1*− (mean S1* bias 62.55% ± 5.88 SEM, t_(29)_ = 2.1362, *p* = 0.0412, g = 0.3900) at about the same rate they chose S1+ over S1− (mean difference − 0.0240, t_(29)_ = 0.8032, *p* = 0.4284, g = 0.0734). S1 and S1* decision biases are normalized by the corresponding S2 decision bias^14^. Error bars show the standard error of the mean. Dots indicate individual subject’s decision bias (n = 30).

Memory guidance in the form of transfer of reward from S2 to S1 should become evident during the decision-making phase. We thus operationalized transfer of reward as the bias to choose stimuli associated with reward (S1+) over those associated with neutral outcomes (S1−). Following Wimmer and Shohamy^14^, we calculated a decision bias score as the likelihood to choose S1+ over S1−, normalized by the mean bias to choose S2+ over S2− to account for variable strength of S2 conditioning (see Methods). Subjects expressed a significant choice bias for S2+ over S2− (Fig. 2, mean S2+ bias 73.89% ± 3.7 SEM, t_(29)_ = 6.4572, *p* < 0.001, g = 1.1482). This suggests that differential conditioning for specific S2+ versus S2− during the preceding conditioning phase was successful. Importantly, subjects showed significant transfer of reward from S2 to original views of S1 (Fig. 2, mean S1+ bias 64.96% ± 5.92 SEM, t_(29)_ = 2.5282, *p* = 0.0172, g = 0.4616), replicating the classical sensory preconditioning effect, including effect sizes reported in previous studies^5,14^. Because the decision bias score measures the likelihood to choose S1+ over S1−, a S1 decision bias larger than chance level shows that transfer of reward occurs within category, but only for the previously associated S1+ stimuli—it does not spread indiscriminately to all S1, as would be expected if sensory pre-conditioning would occur on the level of categories. Reaction times for choosing S2+ and S1+ did not differ significantly (mean difference − 12.08 ms, t_(29)_ = − 0.1100, *p* = 0.9131, g = − 0.0130), indicating that memory guidance for associated stimuli did not require additional processing ressources, but that S2 and S1 were treated equivalently in terms of associated reward.

### Generalization to new S1 views

The previous result gives a first indication that memory-guidance is specific; however, transfer of reward could occur at the pictorial level or at the level of facial identity. To dissociate these possibilities, we assessed subjects’ bias to choose between new stimuli S1+* and S1−*. If memory-guidance of decision making was image-specific, we would not expect a significant choice bias here. Instead, we found that subjects choose new views of reward-associated S1 over new views on non-reward associated S1 (Fig. 2, mean S1* bias 62.55% ± 5.88 SEM, t_(29)_ = 2.1362, *p* = 0.0412, g = 0.3900) at about the same rate they chose original views of reward-associated S1 over original views on non-reward associated S1 (mean difference − 0.0240, t_(29)_ = 0.8032, *p* = 0.4284, g = 0.0734); decision biases for S1 and S1* were highly correlated (r = 0.8713, *p* < 0.001). Furthermore, reaction times for choosing S1 and S1* did not differ significantly (mean difference − 19.12 ms, t_(29)_ = − 0.2474, *p* = 0.8064, g = − 0.221). Together, this suggests that transfer of reward is not image-specific, but occurs at the level of identity, generalizing across views.

Previous research has shown that reward pairing can spread along continuous stimulus dimensions, e.g., when an oriented line is paired with reward, this can spread to lines tilted more than 20 degrees away from the original stimulus^22^. We thus assessed the width of the gradient of generalization for transfer of reward across head orientations (Fig. 1b). A narrow gradient of generalization would be suggestive of some degree of tuning of the S2–S1 association to head orientation, whereas a broad gradient of generalization would imply view invariance. A third possibility is a mirror-symmetric pattern of generalization, whereby reward spreads between profile views, but not between profiles and frontal views. A repeated measures ANOVA (rmANOVA) on S1* decision biases with the factor head orientation revealed no significant effect (F_(2,118)_ = 0.3605, *p* = 0.6981, η^2^ = 0.0029). Furthermore, there was no significant difference between the mirror-symmetric and non-mirror-symmetric conditions (t_(29)_ = 0.6493, *p* = 0.5212, g = 0.1706). This suggests that transfer of reward is neither head orientation-dependent nor follows a mirror-symmetric pattern but is view invariant (across the orientations tested here).

### Controls for dimensions other than identity and head orientation

Faces are high-dimensional stimuli, and although we controlled for or equated many basic (luminance) and higher-level dimensions relevant to face processing (gender, expression, race, trustworthiness), it is thinkable that dimensions other than identity and head orientation drove decision bias and/or generalization. We thus assessed whether two additional dimensions, likeability and memorability, which are known to affect memory, impacted our results.

*Likeability* is a subjective dimension that varies idiosyncratically between participants; more likeable faces are more easily recognizable^23^. To assure that choice bias during the decision-making phase was not driven by differences in likeability between faces (instead of associated reward), we determined the most neutral faces from a larger set of stimuli for each individual subject before the association phase and used only those images in our experiments (see Methods). To assess residual effects of likeability despite titration, we correlated likeability as assessed before learning and original S1 decision bias per image per subject. The mean Pearson correlation coefficient was r = 0.1120 (SD = 0. 3815) and not significantly different from 0 (t_(29)_ = 1.6075, *p* = 0.1188, g = 0.2935). In fact, learning had no significant effect on likeability of S1 [rmANOVA with factors reward (+, −) and time (pre, post), all *p* > 0.43], as in previous studies^14^, and correlations between decision bias and likeability remained close to 0 and non-significant after learning (r = 0.0101, SD = 0.3830, t_(29)_ = 0.1451, *p* = 0.8857, g = 0.0265). We also obtained likeability ratings for S1* after the decision-making phase. A rmANOVA with factors reward (+, −) and view (original, novel) revealed no significant effects (all *p* > 0.17), while likeability of original and novel views were highly correlated (rewarded original & novel views r = 0.5193 ± 0.3544 SD, t_(29)_ = 6.7939, *p* < 0.001, g = 1.2404). Together, this suggests that decision making was driven by reward association, and not by intrinsic likeability of the stimuli.

**Figure 3:**
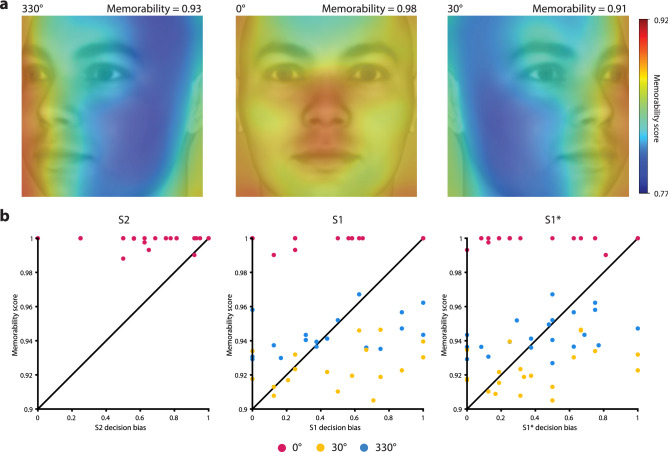
Memorability and decision bias. (**a)** Heatmaps of image-computable memorability. Memorability was generally high, as would be expected for faces, but did show a dependency on head orientation (mean memorability score above the images). Memorability was generally highest for frontal views (mean difference to profile views 0.0658, t_(70)_ = 21.5698, *p* < 0.0001, g = 5.3345). Faces are taken from “25 White Faces Manipulated on Trustworthiness (Steps of 1 SD)” data set from the Social Perception Lab (http://tlab.princeton.edu/databases/), and are reproduced with permission. (**b)** Because memorability depends on view (one-way ANOVA, F_(2,69)_ = 429.1962, *p* < 0.0001, η^2^ = 0.9256), but decision bias does not, there were no statistically significant correlations between the average S2 (r = − 0.0384, *p* = 0.8587), S1 (r = 0.0131, *p* = 0.9268), and S1* (r = 0.0842, *p* = 0.5190) decision bias for an image and its memorability score.

*Memorability*, i.e., the likelihood that a stimulus is later remembered or forgotten, is partially but strongly driven by image features^24,25^. To assess whether image-computable memorability determined choice bias or generalization, we used a deep neural network (MemNet^26^, see Methods) to compute memorability scores and correlated them with the average choice bias for S1 and S1* images. We found that memorability scores across all images were strongly driven by head orientation (Fig. 3a, one-way ANOVA, F_(2,69)_ = 429.1962, *p* < 0.0001, η^2^ = 0.9256), with higher memorability for frontal than for profile views (mean difference 0.0658, t_(70)_ = 21.5698, *p* < 0.0001, g = 5.3345). We found no such view-dependence of average decision bias for the subset of S1 stimuli (one-way ANOVA, F_(2,49)_ = 0.2393, *p* = 0.7881, η^2^ = 0.0097), and there was no correlation between memorability scores and S1 decision bias (Fig. 3b, r = 0.0131, *p* = 0.9268). Similarly, S1* decision bias across images did not depend on head orientation (F_(2,58)_ = 0.0387, *p* = 0.9621, η^2^ = 0.0013), and did not correlate with memorability scores (Fig. 3b, r = 0.0842, *p* = 0.5190). Thus, associative memory guidance and generalization thereof did not depend on image-driven memorability.

### Role of exposure

Previous research suggests that transfer from S2 to S1 in sensory preconditioning depends on the amount of learning during the association phase, initially increasing with the number of pairings and declining thereafter^27,28^. We thus assessed whether the amount of training affected memory-guided decision making and generalization. To this end, we compared S2, S1 and S1* decision bias of the group which was trained for one block (12 trials per pair) of associative learning and the group that was trained for four blocks (48 trials per pair). We found no difference in decision bias for S2 (t_(28)_ = 0.7451, *p* = 0.4624, g = 0.2647), S1 (t_(28)_ = 0.1205, *p* = 0.9049, g = 0.0428), nor S1* (t_(28)_ = 0.4830, *p* = 0.6329, g = − 0.1716). Thus, transfer of reward through sensory preconditioning and generalization across views were not significantly affected by the amount of associative learning in our study. We note, however, that statistical power to detect small effects was low given the sample size for these comparisons.

### Role of awareness

Previous studies have shown that associative learning of face pairs does not lead to awareness of these associations^29^ and that memory guidance occurs in the absence of explicit memory about the associations that form the basis of this guidance^14^. Similarly, we found that subjects were not better than chance in their explicit judgement whether two faces formed a pair when they actually did (mean difference − 3.68%, t_(29)_ = − 1.44, *p* = 0.1606, g = − 0.2629). Across subjects, there was no consistent correlation between the percentage at which two faces were recognized as belonging to a pair and the associated S1 decision bias (mean r = 0.2031, SD 0.8979, t_(29)_ = 0.7718, *p* = 0.4465). Pairs that were more often recognized than chance did not show stronger S1 decision bias than those that were recognized at chance level or below (mean difference − 0.0389, t_(88)_ = − 0.4488, *p* = 0.6547, g = − 0.0980). This all indicates that memory guidance occurs in the absence of awareness, suggesting that the effect of memory on decision-making is not only spontaneous, but also automatic.

However, when we exchanged S1 head orientation during the explicit memory test, subjects were less likely than chance to respond that S1 and S2 belonged to a pair (mean difference − 9.08%, t_(29)_ = − 4.4159, *p* < 0.001, g = − 0.8062). They also correctly recognized at above chance levels that faces did not belong together when we re-shuffled original face images into new S1–S2 pairs (mean difference 9.44%, t_(29)_ = 2.6810, *p* = 0.0120, g = 0.4895). This suggests that although subjects did not explicitly recognize pairs in their correct configuration, they had access to other information that allowed them to distinguish new, unfamiliar from old, familiar stimulus pairs.

### Role of ability

Memory guidance of value-based decision making about faces may also be affected by an individual’s ability to maintain and utilize information about faces in memory. To assess this possible relationship, we used the CFMT^21^ as an independent measurement of face episodic memory ability. Mean performance on the CFMT was 83.29 ± 11.70% (range 57–99%), with a mean score of 60 ± 8.42 SD (range 41–71). All scores fell within the normal range for participants in Germany^30^. Across subjects, face episodic memory performance (in percent) as assessed by the CFMT was not significantly correlated with S2 (r = 0.3315, *p* = 0.0735), S1 (r = − 0.1417, *p* = 0.4550), or S1* (r = − 0.0946, *p* = 0.6191) decision bias. Hence, memory-guidance of value-based decision making for faces is subject to different capacity constraints than face episodic memory.

## Discussion

We find that associative memory-guidance of value-based decision making is sufficiently specific to occur within a category. This specificity clearly has adaptive value for categories with relevant within-category variation, such as faces depicting different identities. At the same time, we find that memory guidance is not narrowly bound to pictorial information present during memory encoding but generalizes spontaneously across new stimuli (head orientations) to a higher level of abstraction, here facial identity. Thus, memory-guidance of value-based decision making seems to strike a balance between specificity and generalization by operating on a meaningful level of representation that neither over- nor underspecifies associative relationships in the context of obtaining reward.

Previous research using unidimensional stimuli such as oriented lines^22^ has shown that stimulus generalization in conditioning follows a gradient along this dimension that depends on the perceptual similarity between the conditioned and new stimuli. We extend this research in two ways: 1. By showing that generalization also occurs for associated stimuli that were never rewarded themselves; and 2. by showing that generalization of association can occur along a relatively abstract dimension such as head orientation that entails many pixel-level differences between images that are unified by the three-dimensional transformation of the underlying identity, even across large angular distances (here up to 60°). These two levels, pictorial and abstract, cannot be dissociated when using simple, unidimensional stimuli such as oriented lines or pure tones.

Research in humans and other animals has implicated category-selective visual cortex, hippocampus, orbitofrontal cortex, and striatum in the generalization processes that enable transfer of reward and memory-guided decision making. While visual cortex provides sensory information that can be associated with reward by the striatum, the hippocampus in particular has emerged as a key node in generalization: lesions to the hippocampus^31^ as well as hippocampal input^32^ and output^33^ structures disrupt sensory preconditioning. It is thought that the hippocampus contributes to associative generalization representing higher-order relationships among discrete items^34^, i.e., S1 and S2, a role that it may share with orbitofrontal cortex^35^, and that may rely on reactivating representations in visual cortex^14^. Furthermore, the hippocampus participates in similarity-based stimulus generalization, such as from S1 to S1*, through connectivity with the striatum^22,36^. In rodents, generalization of memory representation via the hippocampus is further modulated by the nucleus reuniens thalami^37^. Interestingly, many of these structures contain face-selective neurons: view-invariant representations that could plausibly support generalization exist in higher visual cortex^17^, orbitofrontal cortex^38^, and the hippocampus^18^, while intermediate stages of cortical face processing^17^, orbitofrontal cortex^38^ as well as the striatum^39^ harbor view-specific representations. This suggests that generalization from S2 to S1* as we found in our study may involve invariant representations in the hippocampus and/or higher order visual cortex.

Generalization from S2 to S1 has been suggested to take place at encoding, i.e., when S2 is paired with reward, the previously established S1–S2 association is retrieved and leads to integrated encoding of S1 with reward in memory. This view is supported by functional magnetic resonance imaging studies showing that hippocampal activity during encoding is related to S1–S2 generalization at test^14^ (also see^40,41^). Alternatively, retrieving and integrating S1 could also occur at the time when the decision is taken^35,36^. This debate parallels the question when generalization from S1 to S1* takes place, which could also occur at encoding, e.g., by encoding memories that associate reward with view-invariant representations, or at retrieval, relying on a mechanism that accesses memories in an invariant fashion^42^. Alternatively, generalization could involve forgetting of entire stimulus dimensions, such that subjects remember only identity but not head orientation information. These scenarios cannot be dissociated easily based on behavioral data alone.

Transfer of reward from S2 to S1 and generalization from S1 to S1* did not occur equally for all subjects and for all stimulus pairs, as in previous studies^14^. We aimed to minimize inter-stimulus variability by equalizing low-level stimulus features, valuation, and other social dimensions inherent to faces, and we found no correlation between generalization and image-driven memorability. Although we cannot control every possible stimulus dimension, this still suggests that a significant part of the observed variability stems from inter-individual differences. While we can rule out differential face episodic memory ability as a driving source of variability, previous research has suggested inter-individual differences in neural coupling between frontal cortex and the hippocampus as a possible source^19^. Studies that have reported more sizeable generalization effects from S2 to S1 differ from our and similar^14^ studies in that they used a much smaller number of stimuli^12^ or gambling tasks that involved losses^43^, whereas we only used neutral outcomes and gains. Generalization involving appetitive and aversive outcomes may involve different mechanisms^44^, and may have interacted with inter-individual variability by triggering risk-aversive behaviours and/or differential sensitivity to losses and gains. Unraveling the factors that determine variability in transfer awaits further investigation.

In summary, we find that value-based decision-making is guided by an adaptive memory system that can spontaneously and automatically provide both highly specific information about individuals and generalize across other dimensions, here head orientation. Future research should investigate whether this flexibility is also present for stimuli whose dimensions do not entail intrinsic ecological relevance.

## Materials and methods

### Subjects

Data were acquired in 34 subjects (mean age 26.5 ± 5.5 SD years, 19 females, all right-handed) after they provided written informed consent. All subjects reported normal or corrected-to-normal vision, no neurological or psychiatric disorders nor taking psychoactive drugs. Subjects were paid €8 per hour and could earn up to €5 reward for their performance in the reward and decision phase. Four subjects were excluded from data analysis because they did not show learning of at least one of the conditioned stimuli (final n = 30, mean age 26.9 ± 5.7 years, 17 females, all right-handed). Sample size was based on previous studies^14^. All procedures were in accordance with the Helsinki Declaration of the World Medical Association and approved by the Ethics Committee of the University Medical Center Göttingen (protocol number 29/8/17).

### Stimuli

For our main experiments, we used computer-generated faces (FaceGen, v3.1, Singular Inversions) available online from the Social Perception Lab (http://tlab.princeton.edu/databases/), specifically the dataset “25 White Faces Manipulated on Trustworthiness (Steps of 1 SD)”, which includes maximally distinct Caucasian male identities with no hair^45,46^. From this dataset, we selected 24 identities that were neutral on the trustworthiness dimension (0 SD) and showed a neutral facial expression. For each face, we extracted three head orientations—0°, 30° and 330°—a total of 72 images. To control low-level image properties, each image was converted to grayscale and luminance-equalized using the SHINE toolbox^47^ in Matlab (R2018a, The Mathworks). Mean luminance of faces was 50.05 cd/m^2^. All stimuli were displayed against a uniform gray background (53.35 cd/m^2^).

### Overall procedure

To study transfer and generalization in memory-guided decision making, we employed a modified version of a previously established sensory preconditioning paradigm^14^. The paradigm involves three main phases: association, reward and decision. During the association phase, participants were exposed to pairs of faces in order to establish associations between stimuli S1 and S2. During the reward learning, participants underwent classical conditioning, with half of S2 stimuli followed by monetary reward (S2+), and the other half by a neutral outcome (S2−). No S1 stimuli were shown in this phase. During decision-making, participants were asked to select which of two stimuli, S1+ vs. S1− or S2+ vs. S2−, was more likely to lead to a monetary reward. In addition, we obtained likability ratings before the association phase and after the decision-making phase, a test of awareness of stimulus associations, and the CFMT as a test of face episodic memory capacity. All stimuli were presented on a gamma-corrected LCD monitor (VIEWPixx /EEG, resolution 1920 × 1080 px) at a refresh rate of 120 Hz. Subjects viewed the screen from a distance of 65 cm. All experiments were conducted in a sound-attenuated, darkened cabin (Desone Modular Acoustics). Stimulus delivery and response collection were controlled via Presentation (v19.0, Neurobehavioral Systems). The experiments took about 1 h in total.

### Likability ratings and stimulus selection

Individual preference for face stimuli could vary among subjects and thus bias their choices in the decision phase. To accommodate this, we selected the 12 most neutral identities from the dataset based on the individual likability ratings before the association phase for each subject. Participants rated 24 different neutral facial identities using a continuous visual analogue scale with three anchors, from “I don’t like it” to “Neutral” to “I really like it”. The visual analogue scale and the image (6.7 × 11.5 dva) were presented simultaneously, and subjects were instructed to respond by selecting the corresponding location on the scale via a mouse click. The stimulus remained on the screen until the response was obtained. Because likeability as well as other high-level facial dimensions such as trustworthiness are known to be highly consistent across rigid rotations of the head in the horizontal plane^48–50^, each of 24 identities was presented only in one view: 16 were presented in a frontal view (0°), four at 30° and four at 330°. Each identity was presented twice, resulting in a total of 48 trials, taking about 4 min.

To select neutral stimuli per subject, we first normalized the ratings by their standard deviation, and selected eight of the most neutral frontal faces, two of the most neutral 30° faces, and two of the most neutral 330° faces.

Likability ratings for the selected images were collected again after the decision-making phase in order to assess effects of conditioning and transfer. Here, we also presented additional views that had been used as S1* during the decision-making phase (see below).

### Association phase

For the association phase, we pseudo-randomly assigned the pre-selected neutral face images to six pairs (S1–S2). In each pair, S2 was always presented as a frontal view (0°). S1 was shown at 0° (2), 30° (2), or 330° (2). To form associations between the stimuli within pairs, we used a statistical learning paradigm^20,51^. Subjects passively viewed the faces in temporal sequences. To induce statistical structure, the sequence of pairs was arranged such that transition probabilities within pairs (i.e., between stimuli) were 100%, while transition probabilities between pairs (i.e., between trials) were at minimum and balanced across pairs. Each pair appeared 12 times per block. Images (8.4 × 14.4 dva) were presented at fixation for 250 ms each, with a 250 ms inter-stimulus interval (ISI) and a 617 ms inter-trial interval (ITI). The ITI was longer than the ISI to aid implicit pairing^7^. The duration of one block was approximately 2 min. Subjects were not instructed about the pairing of the stimuli but only to fixate on a blue fixation dot. To assure that subjects were paying attention to the images, they performed a 1-back repetition detection task, i.e., they had to report an infrequent (12 per block) immediate repetition of an identical image (i.e., identical identity and view) by means of a button press on a standard keyboard.

To assess whether the amount of exposure during the association phase had an effect on memory guidance, one group of subjects (n = 15, mean age 26.6 ± 7.15 years, 8 female) was trained for one block (12 trials per pair) and one group (n = 15, mean age 25.1 ± 3.85 years, 9 female) for four blocks (48 trials per pair).

Performance on the cover task was well above chance level. In the group receiving one block of training, mean accuracy was 91.11% ± 2.63 (SEM; significantly above chance performance, t_(29)_ = 15.6254, *p* < 0.001) and the average reaction time 519.23 ms ± 28.93 (SEM); in the group receiving four blocks of training, average accuracy was 96.63% ± 0.57 (SEM; significantly above chance performance, t_(29)_ = 81.1336, *p* < 0.001) and average reaction time 563.02 ms ± 22.06 (SEM).

### Reward phase

During the reward phase, subjects learned the association between value and S2 stimuli through classical conditioning. Specifically, they learned to associate one of the S2 stimuli per view category with a monetary reward (S2+) and the second one with a neutral outcome (S2−). To this end, presentation of S2+ stimuli was followed by a picture of a €1 coin; presentation of S2− stimuli was followed by a white square. Each S2 stimulus was presented 16 times per block, in pseudo-random order. Because conditioning is more robust with reinforcement rates below 100% percent^52,53^, only 13 of 16 S2+ trials were followed by reward (81%). For the S2−, neutral outcome always followed the stimulus. Subjects were instructed to press corresponding buttons on a keyboard in response to the €1 coin and the white box, respectively. They were also informed that correct responses would earn them actual reward and that they may notice predictive associations between faces and the subsequent outcomes. S2+/S2− stimuli and rewards were shown for 1 s, with an ISI of 1 s and an ITI between 0.5 and 2 s. Conditioning proceeded over two blocks of 96 trials and lasted approximately 16 min in total. No S1 were shown.

### Decision-making phase

In the decision-making phase we assessed whether subjects used memory of the previously associated stimuli to guide their value-based decisions. To this end, we employed a 2AFC task. On each trial, two identities in the same view were presented simultaneously. The question “Which face is likely to lead to winning €1?” appeared above the images. Subjects were informed that they would not receive feedback but that they would earn reward through correct responses. Subjects selected one of the images with the respective button press. Both stimuli remained on the screen until the subject made a choice.

Memory guidance in this task is evident when subjects choose a previously rewarded (or reward-associated) stimulus over a non-rewarded stimulus on a given trial. Subjects were presented with S2+ vs. S2− and S1+ vs. S1− stimuli with the same head orientation (e.g., frontal vs. frontal / 30 vs. 30 degrees / 330 vs. 330 degrees). In addition, we presented S1+ vs. S1− with new head orientations that had not been shown during the association phase to assess whether memory-guidance generalized to new views of the same identities. E.g., if subjects had learned to associate a frontal S1+ with S2+, they would now also be shown S1+ at 30 and 300 degrees, respectively, vs. S1− at the same head orientations. Each choice pair was repeated 8 times for original S1+ vs. S1− and 4 times for S2+ vs. S2− and S1+* vs. S1−*, respectively. Stimuli were randomly permuted on the left and right side for each repetition, resulting in an equal amount of presentations on both sides. The decision phase consisted of 60 trials in total and the data was acquired in one block of approximately 5 min duration.

### Explicit memory test

To determine whether subjects developed explicit knowledge of S1–S2 associations, or whether they were unaware of these associations, we obtained explicit judgements of association for all exposed pairs as well as foils constructed by shuffling the learned S1–S2 pairs into new combinations; in addition, we also obtained judgements of associations for S1–S2 pairs where the head orientation of S1 was changed. As during the association phase, subjects were presented with S1 and S2 in temporal succession. Stimuli were presented for 250 ms each, with a 250 ms ISI. After each pair, participants were asked whether the two faces they just saw belonged to the same pair. Each pair was presented 4 times in its original configuration, twice with new S1 head orientations (once per head orientation), and once in a new S1–S2 configuration. 84 trials were split into two balanced blocks. The explicit memory test took approximately 4 min to complete.

### Cambridge face memory test (CFMT)

To assess subjects’ episodic face memory ability we carried out the Cambridge Face Memory Test (CFMT)^21^. This test measures a compound of face perception and face episodic memory ability and has been shown to differentiate normal from prosopagnostic face recognition ability. In brief, the CFMT test consists of four parts: (1) a practice phase during which participants learn how to respond during the test; (2) testing recognition of familiar faces right after seeing them (image recognition); (3) testing recognition of familiar faces among novel faces; 4) testing recognition of familiar faces among novel faces with the Gaussian noise added to the images. Performance on the four parts is combined into a face recognition score, which can be expressed in percent.

### Image-computed memorability

We obtained image-computable memorability scores for the stimuli used in our experiments using MemNet^26^ estimates. MemNet is a convolutional neural network trained to estimate image memorability on a large-scale dataset of 60,000 natural images (LaMem)^26^, publicly available at http://memorability.csail.mit.edu. Each image in this dataset is associated with a memorability score based on human performance (corrected for any delay between successive presentations in the human experiments). After training, MemNet estimates visual memorability of natural images near the upper bound imposed by human performance: MemNet estimates reach r = 0.64 rank correlation with mean human-estimated memorability, while the upper bound of consistency between human scores has a rank correlation of r = 0.68.

Memorability scores were obtained using the network weights reported in Khosla et al.^26^ and publicly available at http://memorability.csail.mit.edu/download.html. We used an implementation of this network in PyTorch^54^ and corresponding weights^55^. Before passing the stimuli into MemNet, we preprocessed the stimuli as follows: we resized the stimuli to 256 × 256 pixels (with bilinear interpolation), subtracted the mean RGB image intensity (computed over the dataset used for pretraining of the network), and then center cropped the stimuli to a size of 227 × 227 pixels. Each center cropped image was passed through MemNet and the raw memorability prediction was obtained for each input image. Then, this raw prediction was linearly transformed to obtain the estimated memorability score:

$$Memorability \;Score=\mathrm{min}\left(\mathrm{max}\left(\left(output-mean\; pred\right)*2+additive\; mean, 0\right), 1\right)$$

Following Khosla et al.^26^, we set mean_pred = 0.7626 and additive_mean = 0.65. Correlations between memorability scores and decision biases were assessed using Pearson’s correlation coefficient.

In addition, we obtained a heatmap of memorability for all preprocessed images. To generate memorability heatmaps, first we converted MemNet into a fully convolutional network as described in Long et al.^56^. To this end, we converted the three top fully connected layers of MemNet (fc6, fc7, fc8_euclidean) to three convolutional layers of size 6 × 6, 1 × 1, and 1 × 1, respectively. This fully convolutional network can now be applied to images of arbitrary sizes to generate different sized memorability heatmaps. We used the following six input image sizes: 227 × 227 pixels (generates 1 × 1 heatmap), 281 × 281 pixels (generates 3 × 3 heatmap), 339 × 339 pixels (generates 5 × 5 heatmap), 451 × 451 pixels (generates 8 × 8 heatmap), 563 × 563 pixels (generates 12 × 12 heatmap), and 675 × 675 pixels (generates 15 × 15 heatmap). Then, we rescaled all output heatmaps to the original size of the cropped input images (227 × 227) and averaged them to obtain the final memorability heatmap per stimulus. By converting MemNet into a fully convolutional network (through applying the convolutional operations instead of linear operations of the original three fully connected layers), we use it for segmentation, which produces a memorability heatmap for segmented features of an input image. As a result, the mean of this final heatmap is not generally equal to the memorability score of the input image.

### Statistical analyses

#### Decision bias

Decision bias was operationalized as the subjects’ tendency to prefer the rewarded stimulus (S2+ or S1+ or S1+*) over the neutral one (S2− or S1− or S1−*). Following Wimmer and Shohamy^14^, we computed a decision bias score for each S2+ and S1+ by averaging the subject's preference for the single stimulus over all of its presentations, followed by divisive normalization by the subject’s average S2+ bias to account for variability of learning in the conditioning phase. Also following Wimmer and Shohamy^14^, normalized S1+ decision bias greater than 100% was set to 100%.

#### Likability ratings

For the pre- and post-likability ratings, we normalized each subject’s data by the respective standard deviation across ratings, and then averaged the standardized scores (2 scores per image). Outliers were identified as those images that exceeded 3.5 × the median absolute deviation and were replaced by NaN. Because the distribution of normalized likeability ratings was skewed, we further transformed the data via the inverse hyperbolic sine transform^57^ before entering them into a rmANOVA. For correlation analyses between decision bias and likability Pearson’s correlation coefficients were calculated between the mean normalized likability per image and its associated decision bias per subject. These coefficients were transformed into Fisher’s z-scores via the inverse hyperbolic tangent function (*atanh* in Matlab) for tests of significance, and back-transformed via the hyperbolic tangent function (*tanh* in Matlab) for reporting means and standard deviations.

#### Explicit memory test

To assess the correlation between the percentage of correctly recognized face pairs in the explicit memory test and the decision bias for S1 stimuli in these pairs during the decision making phase, we computed Pearson’s correlation coefficients between these two measures across rewarded pairs per subject. We then transformed the correlation coefficients into Fisher’s z-scores via the inverse hyperbolic tangent function (*atanh* in Matlab) for tests of significance, and back-transformed via the hyperbolic tangent function (*tanh* in Matlab) for reporting means and standard deviations. When correlation coefficients could not be computed because at least one of the variables was constant, r was set to 0 before transformation. To avoid infinite z-scores, correlation coefficients > 0.999320 were set to 0.999320 and correlation coefficients < − 0.999329 were set to − 0.999329. This puts the ceiling/floor to z = 4.

#### Measures of effect size

For all *t*-tests, we computed Hedges’ *g* and for all ANOVA partial *η*^2^ as measures of effect size using the Matlab toolbox Measures of Effect Size (v 1.6.1; https://github.com/hhentschke/measures-of-effect-size-toolbox/)^58^.

All analyses were carried out in Matlab (R2018a, The Mathworks, Inc.) unless otherwise noted.

## Data Availability

The datasets generated during and/or analyzed during the current study are available from the corresponding author on reasonable request.

## Code availability

Code generated for the current study is available from the corresponding author on reasonable request.
